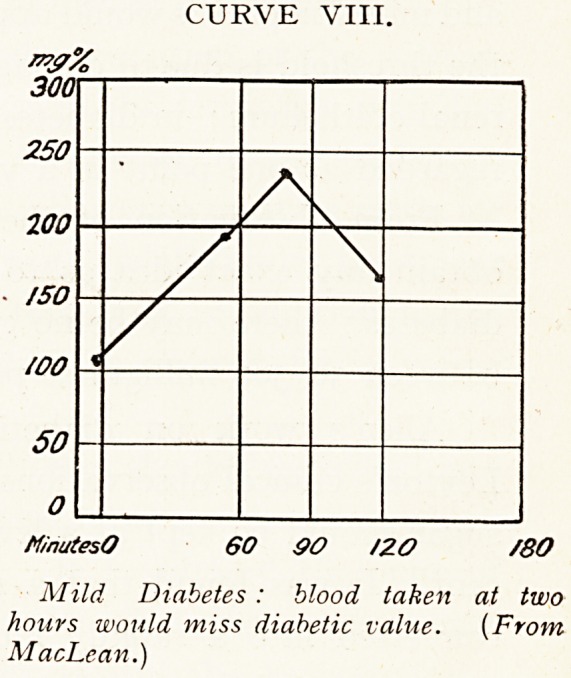# On the Diagnosis and Treatment of Diabetes Mellitus, with Special Reference to the Usages of Insulin

**Published:** 1923-10

**Authors:** A. T. Todd

**Affiliations:** Clinical Pathologist, Bristol Royal Infirmary; Demonstrator of Pathology, University of Bristol


### ON THE DIAGNOSIS AND TREATMENT OF DIABETES MELLITUS, WITH SPECIAL REFERENCE TO THE USAGES OF INSULIN.

BY

A. T. TODD, M.B., Ch.B. Edin., M.R.C.P. Lond.,

*Clinical Pathologist, Bristol Royal Infirmary; Demonstrator of Pathology, University of Bristol.*

---

THE possibility of a diagnosis of diabetes usually arises on the finding of a reducible substance in the urine ; glucose is but one of these, and some test must be made to differentiate it from the others, of which the most frequent are :—

Uric acid, hippuric acid, salicyluric acid present in salicylate therapy, glycuronic acid present after chloral, etc. ; alkapton, creatinine, lactose during lactation ; pentose—in pentosuria.

A simple means of differentiation is possible in the fermentation test, provided certain precautions are taken :—

(i.) Glucose fermenting bacteria are common in urine ; these bacteria will produce gas from other substances than glucose ; to destroy them the apparatus and the urine must be carefully sterilised by boiling.

(ii.) There may be fermentable substances in the yeast itself ; an aqueous extract should be tested by fermentation of the yeast.

(iii.) Brewer's yeast should be used ; baker's yeast sometimes contains other fungi.

A more accurate method is to prove the presence of the osazone derivative.

Glycosuria having been proved, the next step is to determine whether it is diabetic or not. To this end the examination of the blood sugar is the simplest and most reliable method.

Under normal conditions glucose is present in the blood at a concentration of about 100 milligrams, or 0.1 per cent. The amount varies with the state of health (Curve I.), with emotion, exertion and fatigue, but only within narrow limits. The greatest variation is that which is found to occur shortly after taking food. This is seen in the blood sugar curve, a graph constructed from the sugar values at definite periods before and after a test meal. Normally a short positive wave is found, of definite shape and duration. This is termed the alimentary glycæmia wave (Curve II.).

It is seen that the sugar value rises sharply to 140 or 150 milligrams, it then as rapidly sinks, reaching its fasting level, or a slightly lower one, at 90 minutes from the taking of the meal. The height of the peak is not strictly related to the amount of carbohydrate present in the test meal; the same response may be found after two ounces of lean beef as after four ounces of pure glucose. [1]

The explanation of this alimentary wave is still obscure. It might be suggested that the increase of sugar was due to the temporary lack of pancreatic hormone in the blood-stream, that the pancreas was somewhat sluggish in relation to storage of absorbed carbohydrate. That such an explanation is inadequate is obvious from the facts that :—

(1) The height of the glycæmia is independent of the amount of carbohydrate ingested. [1]

(2) If at the depression of the wave another carbohydrate meal is given there is no second wave, except in diabetes. [1]

(3) Glucose administered per rectum does not produce an alimentary wave. [2] In this case the glucose is not absorbed into the portal circulation. It would appear from this latter observation that the liver is important in the storage and metabolism of glucose. A consideration of the experimental and clinical work undertaken to explain the alimentary glycæmic wave is quite beyond the scope of this article; those interested in this aspect of the subject may find it of value to consult the following references: [1, 2, 3, 4, 5, 6].

The most important features of the normal blood sugar curve are:—

(i.) The peak of the wave lies below the 200 milligram line.

(ii.) Subsidence is rapid; in 90 minutes the fasting level is attained.

It should be noted that the term glycæmia, or amount of sugar in the blood, tends to be restricted to values between 50 and 200 milligrams. If less than 50 milligrams the term hypoglycæmia is used; if more than 200 milligrams the condition is called hyperglycæmia.

In diabetes it is found that the blood sugar curve is abnormal:—

(i.) The fasting sugar level is frequently set high. It may exceed 200 milligrams per cent.

(ii.) The peak of the wave rises above the 200 milligram line. Hyperglycæmia is present at some time.

(iii.) The wave subsides more slowly than in health; the fasting value is rarely attained before 120 minutes.

These features are obvious in Curves III., IV., V. and VIII.

This type of curve occurs in diabetes only, and is recognised as the "diabetic curve." Few of the many conditions associated with glycosuria yield the same definite curve, but there are several apparent similarities which call for mention.

(i.) In the early months of pregnancy, and occasionally during menstruation, glycosuria may be found; in the former condition a border-line curve may be met. [1]

(ii.) In hyperthyroidism and Graves' disease all transitions between normal and diabetic curves are found. This is to be expected when the depressant action of the thyroid gland on the pancreas is remembered. Moreover, diabetes preceding or following Graves' disease is not infrequent.[7] In most cases of hyperthyroidism, however, the glycæmia is normal.[2] If the clinical features plus the blood sugar curve are not sufficiently diagnostic then the following procedures are indicated :—

(*a*) Basal metabolism, the patient should not be starved too long before the analysis is made. In hyperthyroidism the value is consistently high. In diabetes the value is reduced, normal or slightly raised.[8]

(*b*) Depression of the thyroid by X-radiation should result in a normal blood sugar curve, if the condition is due to thyroid activity.

(*c*) Cammidge states that a study of the hydrolysable glucose values may be of help in this differential diagnosis.[9]

(iii.) In parenchymatous nephritis and occasionally in mixed parenchymatous-interstitial nephritis a diabetic curve is sometimes encountered. The great majority of these cases exhibit increased tolerance for sugar, and glycosuria only appears after very large amounts of glucose given by the mouth, seldom until the intake exceeds 100 grammes. Nephritis will rarely give rise to difficulty.[16]

One or two conditions not included in diabetes mellitus, but which give rise to glycosuria, are worthy of consideration :—

Renal diabetes, diabetes innocens. In these cases glycosuria is present, but other symptoms are slight or absent. The blood sugar is found to be normal (Curve VI.). The condition appears to be due to the kidney epithelium allowing glucose to leak into the urine at abnormally low levels—the sluice for glucose is set a little lower. It was suspected that this condition might be an early stage of true diabetes, but cases have been observed for many years, and no such transition has been noted.* Most of the pregnancy and menstruation glycosurias fall into this group.

*Border-line cases.*—Here diagnostic difficulty will be met. Glycosuria may be discovered accidentally, or the family history may suggest the possibility of diabetes. Symptoms are usually absent. The glycæmia is found to be intermediate between the normal and diabetic type, and sugar tolerance is diminished (Curve VII.).

In these cases difficulty is of little importance, for the condition is mild diabetes and should be so treated. If this be not done, there is great risk of true diabetes developing. Graham reports one case in which transition did occur.[3]

*Collection of blood for diagnosis.*—The micro methods are applicable only in cases where the pathologist can attend and deal with the case. For general practice it is advised that about 0.5 to 1.0 c.c. (or one-third to one-half of a drachm) be taken. This amount is most readily obtained from a superficial vein, but multiple puncture of congested finger-tip or ear lobe will frequently yield so much blood; vein puncture, if performed with a sharp, well-polished needle, is probably the less painful method.

Coagulation of the blood alters the glucose value,[11] and also interferes with the technique of glucose estimation. To prevent this a small amount of potassium oxalate should be placed in the blood-receiving tube; this combines with the plasma-calcium and prevents coagulation. The oxalate should be in powder form and sterile; the tube should be provided with a rubber stopper so that the contents can be well shaken to ensure a good solution. There are objections to oxalate,[19] but also a fair number of advantages. About one half-grain of oxalate is quite sufficient.

The estimation of the blood sugar should be made as soon as possible after drawing the blood, for it is found that the

* Parkes Weber's case, 35 years; Sir A. Garrod's case, 9 years.

sugar gradually disappears, and after four hours at room temperature there is frequently a decided loss. If delay is expected, or the specimen is to be sent away by post, then a measured amount of blood is to be added to a known volume of alcohol. Special outfits are procurable for this purpose.

A complete curve is unnecessary, except for a doubtful or border-line case. Two samples, one of the fasting blood and the other at a definite time after the meal, are quite sufficient in about 95 per cent. of the cases.

The fasting blood is taken before breakfast, or at least five hours after the last food or drink. The second specimen should be taken exactly 90 minutes after the meal ; some advise two hours as the best time for taking this specimen, but it will be seen from Curve VIII. that a slight case which was evident at 90 minutes might be missed at two hours.

The composition of the test meal is not of great importance, provided fats be excluded, for fats delay absorption. The simplest meal is 50 gms. (2 ozs.) of glucose in a large tumbler of water or black coffee. Honey may be used instead, and is rather less objectionable, but more must be given. Cheap commercial jam contains about 50 per cent. of glucose, and is readily obtained.

If in either of these specimens a glucose value of 200 milligrams or more is reported the condition can be regarded as diabetes mellitus, with reservations for the exceptions noted above. In a small number of cases the result will be doubtful and the complete curve will be necessary.

Having made a diagnosis, the question of insulin administration will be next considered. Here we are concerned with the dosage and not the indications for insulin.

Methods are now being described which are said to obviate blood analysis ; this appears to be regrettable,

CURVE I.

*Showing effect of fatigue. Curve A is of blood before a holiday. Curve B, blood after holiday. Upper curve is almost of border-line type.* (*Graham.*)

CURVE II.

*Typical normal alimentary wave.*

CURVE III.

*Diabetic Curve.*

CURVE IV.

*Diabetes.*

CURVE V.

*Severe Diabetes.*

CURVE VI.

*Renal Diabetes : blood sugar normal.*

CURVE VII.

*Border-line curve.*

CURVE VIII.

*Mild Diabetes : blood taken at two hours would miss diabetic value.* (*From MacLean.*)

because there is a good deal of experimental and clinical work which seems to show that the blood sugar must be kept at a definite low level if the best results are to be obtained from insulin.[1 2] The new methods mostly consist in observing when glycosuria appears in relation to the amount of carbohydrate in the diet. Under normal conditions sugar appears in the urine when the blood sugar reaches 170–180 milligrams per cent.; this value is termed the threshold value or leak point, for leakage into the urine will then prevent hyperglycæmia from occurring, and so protect the sensitive islet cells of the pancreas.

Now in diabetes the threshold value is usually found to be set at a much higher level than in health, and leakage only occurs at values of 200–300 milligrams per cent. This raising of the threshold value is frequently regarded as being protective, inasmuch as it would tend to retain glucose in the body, but in view of the experimental work by Allen it would be safer to say that such an explanation is not correct. The presence of hyperglycæmia in parenchymatous and mixed nephritis would appear to show that the raising of the threshold is due to damage of the tubular or glomerular renal epithelium; in diabetes the raised threshold should be regarded as one point in a vicious circle (*vide* Note I.).

From observation of the urine only one is unable to obtain any exact idea as to the condition of glycæmia in diabetes; there may be no glycosuria at blood sugar levels between 50–300 milligrams per cent.

Allen's work on diabetic dogs, with Graham's and Leyton's clinical observations, seem to prove that the blood-sugar should be kept at a level of about 130 milligrams per cent. It was found that a mild case of diabetes could be converted into a rapidly fatal one by keeping the blood-sugar at a high level. Histologically easy proof of this was forthcoming. The $\beta$ cells of the pancreas islets were found to have lost their granules, to become vacuolated and show dislocation of the nucleus ; some of the cells were atrophic, being replaced by fibrous tissue. The $\beta$ cells appear to be those concerned with carbohydrate metabolism. There is reason to believe that the cells which are not injured too greatly may recover if hyperglycæmia is prevented.[13] Moreover, in diabetic dogs evidence has been obtained to show that regeneration of islet tissue occurs under favourable conditions.[14]

There is no doubt that considerable temporary improvement will follow the use of insulin in the great majority of cases of diabetes, even though the blood-sugar is not carefully controlled ; the patients put on weight and feel much improved, but such treatment must be regarded as purely palliative and probably harmful in the long run. From the short account of the experimental work, confirmed by several other observers, it appears to be hopeless to expect anything more than temporary amelioration unless the hyperglycæmia is rigidly controlled.

The first step in the dosage of insulin is to draw up a diet based upon the height, age and occupation of the patient. Many diet scales are available, and the formation of a diet is readily accomplished. The occupation of the patient is of considerable importance, and allowance must be made for this. A patient who must work while under treatment requires sufficient calories for this work in addition to the basal requirements. Growth must be allowed for in young subjects.

The carbohydrate of the daily diet should be divided into two equal parts, and given at two meals only, before which the insulin should be injected. It is advised that one of these meals be breakfast, in order that the patient may commence the day with a supply of utilisable carbohydrate. The effects of insulin are maintained for about four and a half hours in the average case ; by giving two doses the effects are spread over the day. Von Noorden recommends that insulin be given four-hourly and three times in the day. This appears to be unnecessary.[15]

The amount to be given is roughly in direct ratio to the intensity of the glycæmia : in an average case five units should be tried first. This dose is given before one of the carbohydrate meals, and after three hours blood is drawn as if for diagnosis. If in this specimen the glucose approaches 150 milligrams the dose is about correct ; if not, a five unit increase should be given until the correct dose is found.

In severe cases the amount necessary will be too large for frequent injection, apart from the cost ; in certain cases any amount will be inadequate. In these cases, and where monetary considerations preclude the optimum, a less valuable dose must suffice.

The dosage required bears no relationship to the amount of carbohydrate in the diet. It was thought that one unit (old style) would be adequate for 2 gms. carbohydrate.[16] This has been disproved.[15] The quantity required depends upon the amount of functioning power of the patient's pancreas.

Having determined the exact dose, the patient is to be kept on this dose and diet for about two weeks to one month. Then the utility of the dose is again checked. When by the action of insulin the carbohydrate store of the body is partly occupied again, it sometimes happens that a somewhat larger dose is needed for efficient storage to continue.[17] The converse is also found. Von Noorden reports that a diminished dose is sometimes equally effective.[15] Consideration of cost alone will not permit neglect of such a possibility.

After this, provided there is no obvious change in the patient and weight is gained in a satisfactory manner, no further blood analysis is necessary until a change of diet is made. It is advised, however, that the dosage be checked at intervals, for though improvement in the patient with need for less insulin is not frequent, yet one or two cases have been already described, and such a change for the better is obviously desirable.

With regard to hypoglycæmia, this is not likely to occur in a patient treated on these lines, provided that a meal is not omitted after the insulin injection. The patient and his attendants should be warned to recognise the symptoms of the condition—slight feeling of faintness, restlessness, flushing or pallor and sweating. The remedy, a small amount of carbohydrate taken from the next meal, is usually to hand.

The outlook for insulin therapy is somewhat obscure. That it has advanced the treatment of diabetes is undoubted, but it may be wise to check the optimism inspired by the lay press, lest carelessness in administration and diminution of a possibly helpful psychic effect lead to some discredit of the remedy. At present insulin is used chiefly in the severe cases which are probably beyond cure. In the milder and border-line cases better results may be expected provided accurate dosage of insulin is maintained, but experience and time alone can decide this part of the problem. It is possible that insulin is not the only substance lacking in the diabetic, for Thiroloix[18] has recently shown that when the pancreas is completely extirpated insulin only delays the fatal result. A pancreatic extract made and experimented with by Cohnheim in 1904 was probably effective owing to the insulin present. It was found that this extract was not wholly adequate to deal with carbohydrate metabolism in diabetes. Other extracts of pancreas containing water soluble substances, not present in insulin, were found to be necessary in addition.[8]

Such reservations are necessary, for there is a tendency to believe that a diabetic + insulin = a normal person. It is unlikely that this conception is correct, and at present it is essential to add correct personal hygiene, diet and glycæmia to the diabetic side of the equation.

NOTE I.—The fixing of the threshold point for glucose is probably not so simple as would appear at first sight. In addition to alteration of the special epithelium by toxic substances, the product of metabolic errors in diabetes, the actual acidity of the blood and the amounts of and relationships between calcium, potassium and sodium ions are of importance. These facts do not militate against the conclusions given above. Those interested should consult the articles dealing with this subject.[20, 21]

### REFERENCES.

[1] Traugott, *Klin. Wchnschr.*, 1922, i. 892.

[2] Rosenberg, "Practische Bedeutung der Alimentären Hyperglykämie Kurve," *Klin. Wchnschr.*, 1922, i. 360.

[3] Graham, "Goulstonian Lectures, 1921," *Lancet*, 1921, i. 951, 1003, 1059.

[4] Cammidge, Forsyth, Howard, "Relation of Liver to the different value of blood," *Lancet*, 1921, i. 1017.

[5] Ditto, "Factors concerning the normal sugar content of blood," *Brit. M.J.*, 1921, ii. 586.

[6] Ambard and Chabanier, "Les Glycémies," *Presse Méd.*, 1921, xxix. 787.

[7] Williamson, "Relation of Graves' Disease to Diabetes and Glycosuria," *Lancet*, 1919, ii. 425.

[8] Woodyatt, *Endocrinology and Metabolism*, "Diabetes," vol. iv.

[9] Discussion on Insulin Therapy, *B.M.J.*, 1923, ii. 446.

[10] *Endocrinology and Metabolism*, "Nephritis," iv. 344.

[11] Falta and Richter Quittner, *Biochem. Ztschr.*, 1919, c. 48.

[12] Allen, *J. Exper. M.*, 1920, xxxi. 556; Allen, *J. Metab. Research*, 1922, i. 1; Leyton, "Diabetes," *Brit. M. J.*, 1923, i. 707; Graham and Harris, "Treatment of Diabetes," *Lancet*, 1923, i. 1150.

[13] Allen, *J. Exper. M.*, *loc. cit.*

[14] Bensley, quoted by MacLeod, *Lancet*, 1923, ii. 591.

[15] Von Noorden and Isaac, "Insulin and Diabetes," *Klin. Wchnschr.*, 1923, ii. 1968.

[16] Medical Research Report on Insulin, *Brit. M. J.*, 1923, i. 737.

[17] Burgess, Osman, Payne, Poulton, "Early Experiences with Insulin," *Lancet*, 1923, ii. 777.

[18] "Experimental Diabetes and Insulin," *Presse Méd.*, 1923, xxxi. 873.

[19] Falta and Richter Quittner, *loc. cit.*

[20] Hamburger, "Permeability in Physiology and Pathology," *Lancet*, 1921, ii. 1039.

[21] Chabanier and Lebert, "Du seuil de sécrétion de glucose par le rein," *Presse Méd.*, 1920, xxviii. 553.

## Figures and Tables

**CURVE I. f1:**
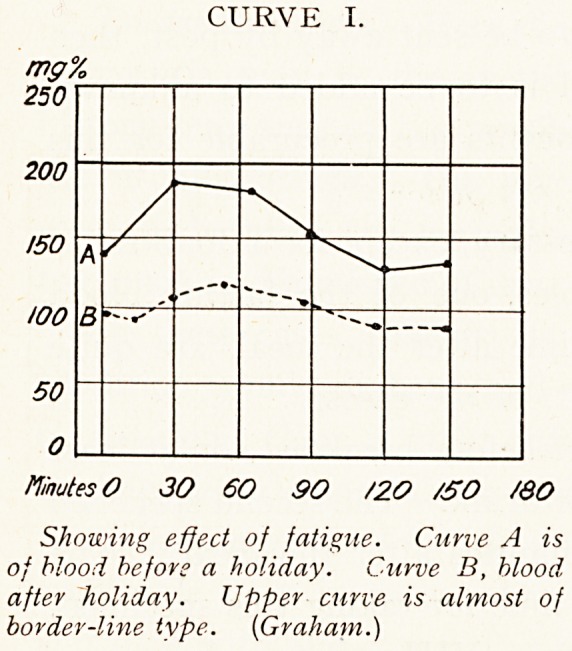


**CURVE II. f2:**
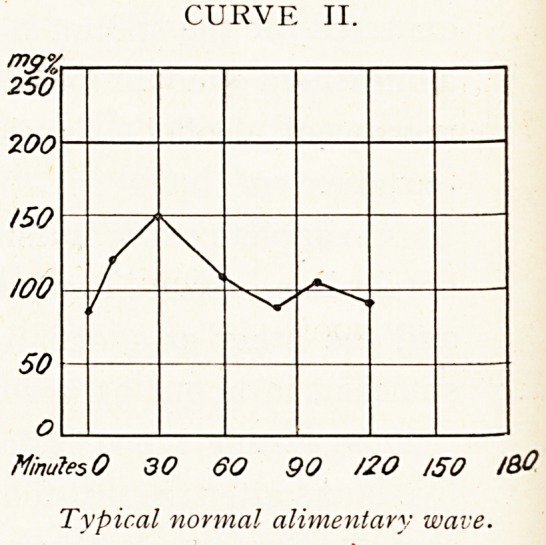


**CURVE III. f3:**
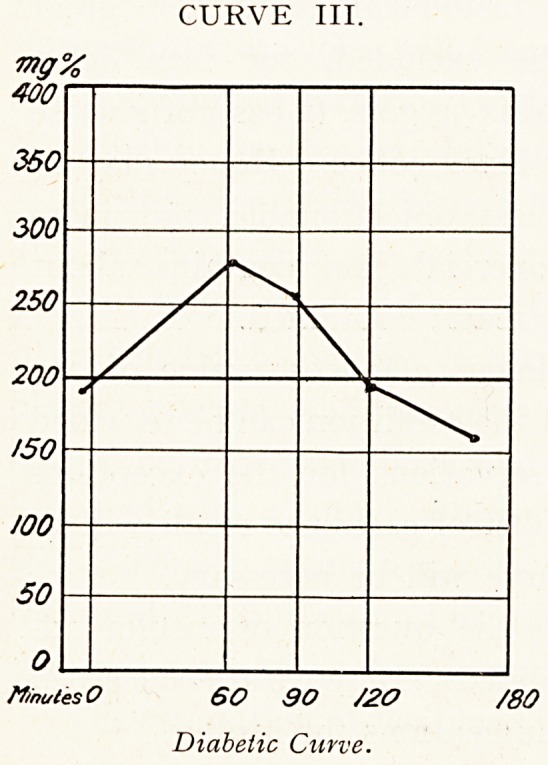


**CURVE IV. f4:**
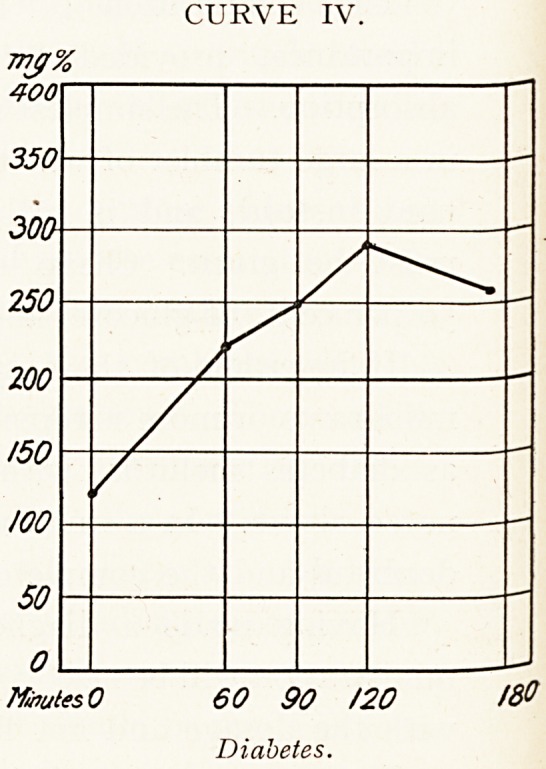


**CURVE V. f5:**
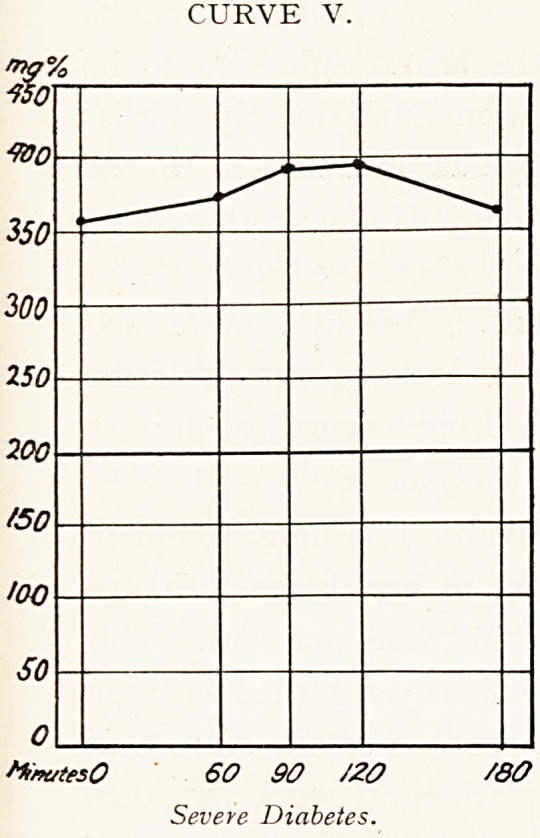


**CURVE VI. f6:**
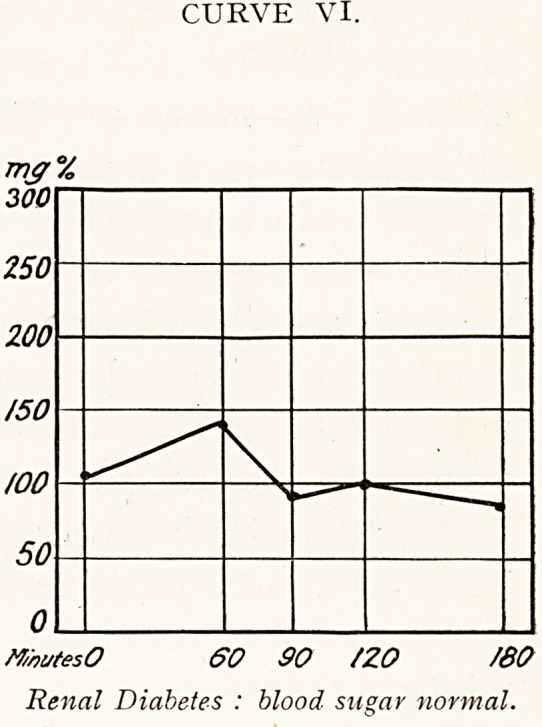


**CURVE VII. f7:**
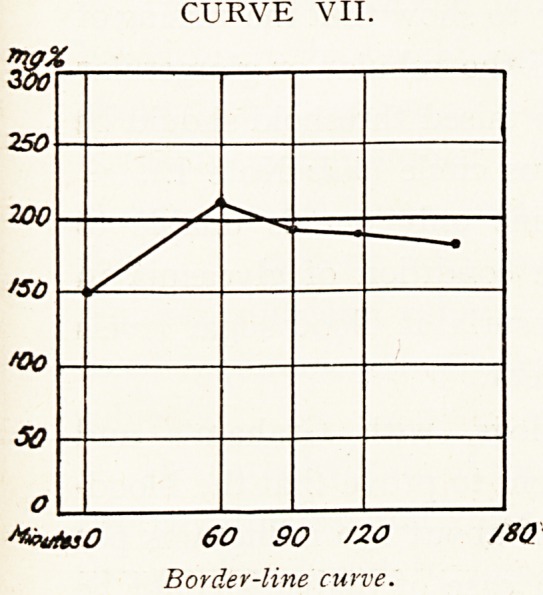


**CURVE VIII. f8:**